# Splenic Gene Expression Signatures in Slow-Growing Chickens Stimulated in Ovo with Galactooligosaccharides and Challenged with Heat

**DOI:** 10.3390/ani10030474

**Published:** 2020-03-12

**Authors:** Elzbieta Pietrzak, Aleksandra Dunislawska, Maria Siwek, Marco Zampiga, Federico Sirri, Adele Meluzzi, Siria Tavaniello, Giuseppe Maiorano, Anna Slawinska

**Affiliations:** 1 Department of Animal Biotechnology and Genetics, UTP University of Science and Technology, Mazowiecka 28, 85-084 Bydgoszcz, Poland; elzbieta.pietrzak@utp.edu.pl (E.P.); aleksandra.dunislawska@utp.edu.pl (A.D.); siwek@utp.edu.pl (M.S.); 2 Department of Agricultural and Food Sciences, University of Bologna, Via del Florio 2, 40064 Ozzano dell’Emilia, Italy; marco.zampiga2@unibo.it (M.Z.); federico.sirri@unibo.it (F.S.); adele.meluzzi@unibo.it (A.M.); 3 Department of Agricultural, Environmental and Food Sciences, University of Molise, Via F. de Sanctis snc, 86100 Campobasso, Italy; siria.tavaniello@unimol.it (S.T.); maior@unimol.it (G.M.)

**Keywords:** GOS, prebiotic, in ovo stimulation, heat, immune response, spleen

## Abstract

**Simple Summary:**

The exposure of animals to excessive heat leads to heat stress, heat stroke, or even death. The first negative effects of heat exposure occur in the gut. The elevated temperature leads to damage in intestinal walls and shifts in the composition of intestinal microbiota. In effect, the gut content (mainly intestinal microbiota and their metabolites) leaks through compromised intestinal walls into milieu of the body. Prebiotics (e.g., GOS—galactooligosaccharides) can be used to mitigate the negative effects of the heat stress in poultry. GOS that are delivered in ovo on day 12 of egg incubation stimulates the development of healthy intestinal microbiota in a chicken embryo. Healthy intestinal microbiota enhances the barrier function of the gut and the immune system. Chickens were originally domesticated in southeast Asia and are therefore genetically adapted to handle high temperatures. However, genetic selection towards performance leads to sensitization to high ambient temperature. In this paper, we studied slow-growing chickens with a reputation for heat resistance. We used in ovo stimulation with the GOS prebiotic that was delivered in ovo to promote healthy gut microbiota. In this manner, we combine genetics and environment to describe a model of heat resistance in poultry.

**Abstract:**

Galactooligosaccharides (GOS) that are delivered in ovo improve intestinal microbiota composition and mitigate the negative effects of heat stress in broiler chickens. Hubbard hybrids are slow-growing chickens with a high resistance to heat. In this paper, we determined the impact of GOS delivered in ovo on slow-growing chickens that are challenged with heat. The experiment was a 2 × 2 × 2 factorial design. On day 12 of incubation, GOS (3.5 mg/egg) was delivered into the egg (n = 300). Controls (C) were mock-injected with physiological saline (n = 300). After hatching, the GOS and C groups were split into thermal groups: thermoneutral (TN) and heat stress (HS). HS (30 °C) lasted for 14 days (days 36–50 post-hatching). The spleen (n = 8) was sampled after acute (8.5 h) and chronic (14 days) HS. The gene expression of immune-related (*IL-2, IL-4, IL-6, IL-10, IL-12p40,* and *IL-17*) and stress-related genes (*HSP25, HSP90AA1, BAG3, CAT,* and *SOD*) was detected with RT-qPCR. Chronic HS up-regulated the expression of the genes: *IL-10*, *IL-12p40*, *SOD* (*p* < 0.05), and *CAT* (*p* < 0.01). GOS delivered in ovo down-regulated *IL-4* (acute *p* < 0.001; chronic *p* < 0.01), *IL-12p40*, *CAT* and *SOD* (chronic *p* < 0.05). The obtained results suggest that slow-growing hybrids are resistant to acute heat and tolerant to chronic heat, which can be supported with in ovo GOS administration.

## 1. Introduction

Acute and chronic thermal stress significantly hinders the growth performance of poultry [1]. This is due to the fact that the feed intake of chickens that are reared in intensive poultry farms is negatively correlated with environmental temperature [2]. The main ancestor of the domestic chicken is red junglefowl (*Gallus gallus*) from the hot jungle climate of Southeast Asia [3]. However, intensive genetic selection for growth and feed efficiency leads to an increased sensitivity of heavy-weight broilers to environmental conditions, including ambient temperature [4]. There is a negative correlation between heat tolerance and growth rate [5]. Fast-growing broilers produce more heat and have a higher heat load [6], which reduces feed intake and growth parameters [7,8,9]. Heat stress (HS) could lead to meat quality issues due to increased ante- and post-mortem glycolytic metabolisms coupled with a reduced protein synthesis and turnover, enhanced fat deposition, and the overproduction of reactive oxygen species [10]. The gastrointestinal tract (GIT) is also very responsive to heat, which alters intestinal microbiota composition [11] and decreases the integrity of the intestinal epithelium [12]. Since exposure to high temperatures is difficult to avoid in intensive production systems, losses in the production and mortality are high [13]. The negative effects of HS in poultry are due to high animal stocking, the insufficient ventilation of poultry houses, as well as geographical factors [14]. Intergovernmental Panel on Climate Change (IPCC) reports clearly show that the climate is warming [15]. Problems related to maintaining optimal temperatures in poultry houses will become more challenging, especially during predicted heat waves, even in moderate climates. HS leads to behavioral, biochemical, and physiological changes. Responses to heat vastly depend on the genetic adaptation of the individual [16] but also on environmental factors, such as intestinal microbiota [17]. Compromised genetic or environmental conditions result in poorer thermoregulation [18].

Heat-resistance depends on the genetic adaptation of the chickens. Native chickens from tropical and sub-tropical regions are more tolerant to high ambient temperatures than fast-growing lines [19,20]. Since they are smaller and lighter and have not been subjected to selective pressure for meat-related traits, they have retained their genetic adaptation to handle high temperatures. Studies on Brazilian breeds (Pelaco and Caneluda) [18] and Egyptian breeds (Fayoumi, Dandarawi, and Sinai) [21] have shown a good tolerance to elevated ambient temperature, manifested by the increased expression of heat shock proteins (HSP). In some countries, native breeds are crossbred with commercial broiler lines to obtain heat-resistant hybrids with good meat production [22]. The hybrids that were used in our experiment are slow-growing free-range chickens that are obtained by crossing a Hubbard RedBro male with a Hubbard JA57 (https://www.hubbardbreeders.com/products/crosses/ja57/) female. These free-range poultry hybrids are distinguished by a good adaptation to a warm climate and a high disease resistance (Federico Sirri, unpublished data).

HS in poultry influences the composition of intestinal microbiota [23], leading to gut dysbiosis [24]. Unstable microbiota impairs the morphology and barrier function of the GIT [25]. Feed additives such as prebiotics support the gut microbiota under stressful conditions [26,27,28]. Prebiotic and probiotic supplementation allow for the maintenance of stable microbiota populations in the gut [29] and prevent “leaky guts” [30]. One of the most efficient ways to improve intestinal microbiota composition in poultry is in ovo stimulation. It allows for the precise introduction of a specific substance directly into the internal environment of the incubating egg. During in ovo stimulation, a prebiotic or synbiotic is injected into the air cell of the incubating egg (in this study, on day 12 of egg incubation) and stimulates the development of indigenous microbiota prior to hatching [31]. The prebiotic supplementation at the embryonic stage supports not only the microbiota development in the growing chickens [32,33,34] but also improves the immune system [35,36], gut morphology [37] and the intestinal barrier function [32]. 

The positive effect of GOS supplementation on the poultry intestinal microbiome has been demonstrated [38]. Galactooligosaccharides are potent prebiotics that exert beneficial effects on intestinal microbiota in chickens. Particularly interesting is the possibility of using in ovo technology for the early stimulation of the microbial communities with GOS [32,39,40]. The molecular data on broiler chickens have indicated that HS triggers systemic immune and stress responses, which are balanced by GOS that are delivered in ovo [41]. It also mitigates the negative effects of HS in broiler chickens on performance traits, including improved growth efficiency, feed efficiency [39], and meat quality [10]. In this study, we focused on chickens with a different genetic background, i.e., slow-growing hybrids. The aim of this study was to assess the impact of GOS that were delivered in ovo on the modulation of the immune-related and stress-related gene expression signatures in the spleens of slow-growing chickens that were subjected to HS. Hereby, we hypothesize that the genetics of slow-growing chickens combined with in ovo stimulation with GOS will contribute to increased HS resistance. 

## 2. Materials and Methods

### 2.1. Ethical Statement

The animal procedures were conducted in compliance with decision of the Ethical Committee in Rome (Italy), decision number 503/2016.

### 2.2. Experimental Trial and Tissue Collection

The experimental material was slow-growing crossbred Hubbard chickens. After 12 days of incubation, 300 eggs were injected with a single dose of 3.5 mg GOS/egg (GOS group) suspended in 0.2 mL of physiological saline into the air chamber. Controls (the C group) were mock-injected with sterile physiological saline (n = 300, volume 0.2 mL/egg). Injection was carried out according to the in ovo procedure [32]. After hatching, the GOS and C groups were divided into two subgroups: maintained in thermoneutral condition (TN) and under the HS condition. In all groups, chicks were sexed and vaccinated against coccidiosis, infectious bronchitis virus, Marek’s disease virus, Newcastle disease, and Gumboro disease, and they received food and water ad libitum. The composition of the diets is presented in Table 1. Male chicks (n = 600, 300 per treatment) were transferred to an environmentally controlled poultry house and divided into 4 groups of 150 chicks/treatment/environmental condition. Each group was composed of 6 replicates of 25 birds each. HS had two forms: acute (on day 36, the temperature in the poultry house was raised to 30 °C for 8.5 h) and chronic (on day 36, the temperature was raised to 30 °C, and these conditions were maintained for 14 days). After that, 8 randomly selected animals from each group with an average body weight were slaughtered and dissected. Fragments of the spleen were collected in the tubes with 3 mL of an RNAlater solution (Invitrogen, Waltham, MA, USA) for RNA stabilization and stored at −80 °C until further processing.

### 2.3. RNA Isolation

Total RNA was isolated from the spleen. Fragments of the spleen tissue were homogenized with the TissueRuptor homogenizer (Qiagen GmbH, Hilden, Germany) in TRIzol^®^ LS Reagent (Ambion/Thermo Fisher Scientific, Valtham, MA, USA). Further steps of RNA isolation and purification were performed with a commercial kit (Universal RNA Purification Kit, EURx, Gdansk, Poland). RNA quality and quantity were evaluated by using electrophoresis in 2% agarose gel and a NanoDrop 2000 spectrophotometer (Scientific Nanodrop Products, Wilmington, NC, USA). 

### 2.4. Quantitative Reverse Transcription PCR (RT-qPCR)

Complementary DNA (cDNA) was synthesized by using the Maxima First Strand cDNA Synthesis Kit for RT-qPCR (Thermo Scientific/Fermentas, Vilnius, Lithuania), following the manufacturer’s recommendations. Obtained cDNA was diluted to 70 ng/μL and stored at −20 °C. RT-qPCR reactions were conducted with a total volume of 10 μL. The reaction mixture included Maxima SYBR Green qPCR Master Mix (Thermo Scientific/Fermentas, Vilnius, Lithuania), 1 μM of each primer (Sigma-Aldrich, Schnelldorf, Germany), and 2 μL of diluted cDNA. Thermal cycling was performed in a LightCycler II 480 (Roche Diagnostics, Basel, Switzerland). Each RT-qPCR reaction was conducted in two technical replicates. Gene expression analysis was performed for two gene panels, which were reported earlier [41]. The first gene panel was associated with the immune response and included the genes: *IL-2, IL-4, IL-6, IL-10, IL-12p40,* and *IL-17*. The second gene panel was associated with stress response and included the genes: *HSP25, HSP90AA1, BAG3, CAT,* and *SOD*. *UB* and *ACTB* were used as reference genes. The sequences of the primers that were used in this experiment are presented in Table 2.

### 2.5. Relative Quantification of Gene Expression and Statistical Analysis

The normalization of the expression levels (Ct − cycle threshold) of the target genes was performed with a geometric mean of the two reference genes (*UB* and *ACTB*). ∆Ct was calculated by subtracting the Ct of the reference genes from the Ct of the target genes (Ct target − Ct reference). All statistical analyses were based on ∆Ct values. The full-factorial study design allowed us to analyze the impact of GOS that were delivered in ovo and different levels of HS on gene expression signatures in slow-growing hybrid chickens. The first statistical model was two-way ANOVA with interaction, in which in ovo treatment and ambient temperature (acute or chronic HS vs. TN) were considered independent variables (factors). In this analysis, the two time-points of tissue collection (day 36—acute HS and day 50—chronic HS) were independently analyzed. The second statistical model included in ovo treatment and HS (acute vs. chronic HS) as factors. In this model, the datasets from acute and chronic HS were combined. In both ANOVA analyses, the factors (or interaction between them) were considered significant at *p* < 0.05, *p* < 0.01, or *p* < 0.001. 

Relative gene expression was calculated with the ∆∆Ct algorithm. In the ∆∆Ct algorithm, a selected a calibrator (control ∆Ct) was subtracted from the ∆Ct of the experimental group. The fold change (FC) of the target gene in the experimental group vs. the control group was calculated according to the formula: 2^−∆∆Ct^ [42]. To visualize the pairwise differences between the treatment groups, the results of the log2 fold change were graphed and compared with a Student’s t-test. The pairwise comparisons were considered significant at *p* < 0.05. The calculations were performed in MS Excel and SAS Enterprise Guide 9.4 (SAS Institute, Cary, NC, USA). Graphs were drawn by using Graph Pad Prism 7 (GraphPad, La Jolla, CA, USA).

## 3. Results

### 3.1. Effects of in ovo Treatment and Thermal Challenge on Gene Expression

The results of two-way ANOVA with interaction, using the first statistical model (GOS vs. C; HS vs. TN; GOS vs. C x HS vs. TN), are presented in Table 3. In acute HS, among the immune-related genes, only *IL-4* responded with differential messenger RNA (mRNA) expression to GOS treatment (*p* < 0.001), temperature (*p* < 0.05), and the interactions between those two factors (*p* < 0.01). GOS treatment modulated the expression of the stress-related genes: *BAG3*, *CAT* and *SOD* (*p* < 0.05). In chronic HS, GOS that were delivered in ovo had immunomodulatory effect on: *IL-2* (*p* < 0.05) and *IL-4* (*p* < 0.001). Temperature significantly modulated the expression of *IL-4* (*p* < 0.01) and *IL-12p40* (*p* < 0.05). GOS treatment, in interaction with the temperature, showed a modulatory effect on stress-related genes: *CAT* and *SOD* (*p* < 0.01). 

The results of the two-way ANOVA that was performed with the second statistical model (GOS vs. C; acute HS vs. chronic HS; GOS vs. C × acute HS vs. chronic HS) are presented in Table 4. In this statistical model, we evaluated the effects of acute and chronic HS on the associated gene expression in the splenic tissue of slow-growing chickens. In ovo treatment had an effect on gene expression of *IL-4* (*p* < 0.001), which was consistent with the results obtained in the first statistical model. HS (acute vs. chronic) changed the gene expression of *IL-2* (*p* < 0.05) and *HSP90* (*p* < 0.05), which was not observed by comparing TN vs. HS (acute or chronic). The interaction of the two factors significantly affected the stress-related genes *CAT* and *SOD* (*p* < 0.05).

### 3.2. Relative Gene Expression Changes in Heat Stress

**Figure 1:**
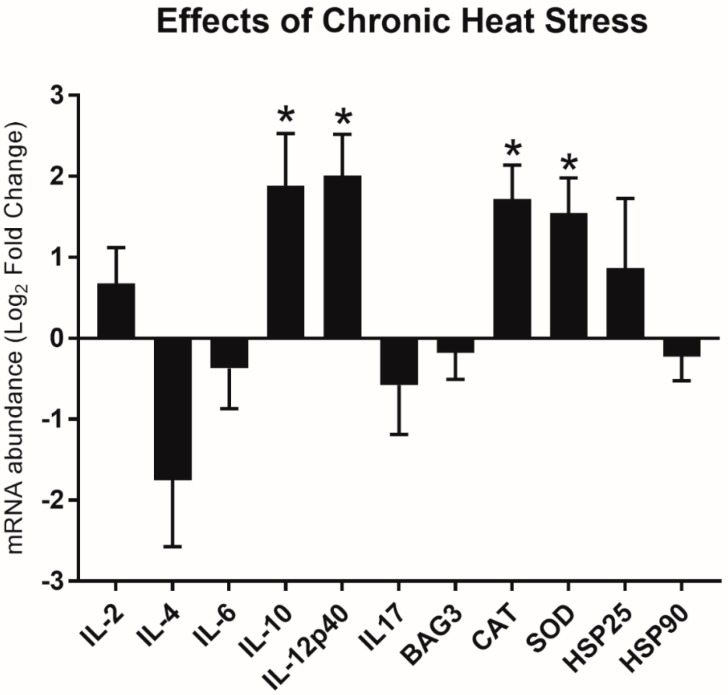
Relative messenger RNA (mRNA) expression of immune-related and stress-related genes in the spleens of slow-growing chickens that were challenged with chronic heat. Gene panel includes: interleukin: *IL-2*, *IL-4*. *IL-6*, *IL-10*, *IL-12p40*, and *IL-17* and stress-related genes: *CAT*, *SOD*, *BAG3*, *HSP25* and *HSP90*. The x-axis shows a list of genes. The y-axis indicate the relative mRNA abundance of the genes after heat challenge (n = 8). Gene expression analysis was carried out with RT-qPCR. qPCR reactions were performed in triplicate. The geometric mean of the *ACTB* and *UB* reference genes was used to calculate delta cycle threshold (dCt) values. The relative gene expression (FC—fold change) was calculated with the delta delta cycle threshold (ddCt) formula and the fold change (FC) was calculated as follows: FC = 2^−ΔΔCt^. FC values were transformed and presented as Log_2_FC. The standard error of the means (SEM) shows distribution of the Ct values. Normalized data (dCt values) of thermoneutral control (mock-injected) and heat-stressed control (mock-injected) groups were compared with a Student’s t-test. Significant differences (*p* < 0.05) are labelled with an asterisk (*). Figures were prepared by using GraphPad Prism 7 (GraphPad, La Jolla, CA, USA).

Short-term (acute) heat (TN-C vs. HS-C) did not affect the gene expression signatures that are associated with immune and stress responses in slow-growing chickens. On the other hand, long-term (chronic) heat activated some immune-related and stress-related genes, presented in Figure 1. Chronic HS up-regulated anti-inflammatory (FC log_2_ of *IL-10* = 1.88, *p* < 0.05) and pro-inflammatory cytokines (FC log_2_ *IL-12p40* = 2.01, *p* < *0.05*). Additionally, oxidative stress was activated in the spleen during chronic HS (FC log_2_ *CAT* gene = 1.72, *p <* 0.01 and FC log_2_ *SOD* gene = 1.55, *p* < 0.05). Surprisingly, the genes encoding chaperones (HSP25 and HSP90) were not activated by either acute or chronic heat (*p* > 0.05).

### 3.3. Effects of GOS Delivered in ovo on Gene Expression Modulation during Acute and Chronic Heat Stress

**Figure 2:**
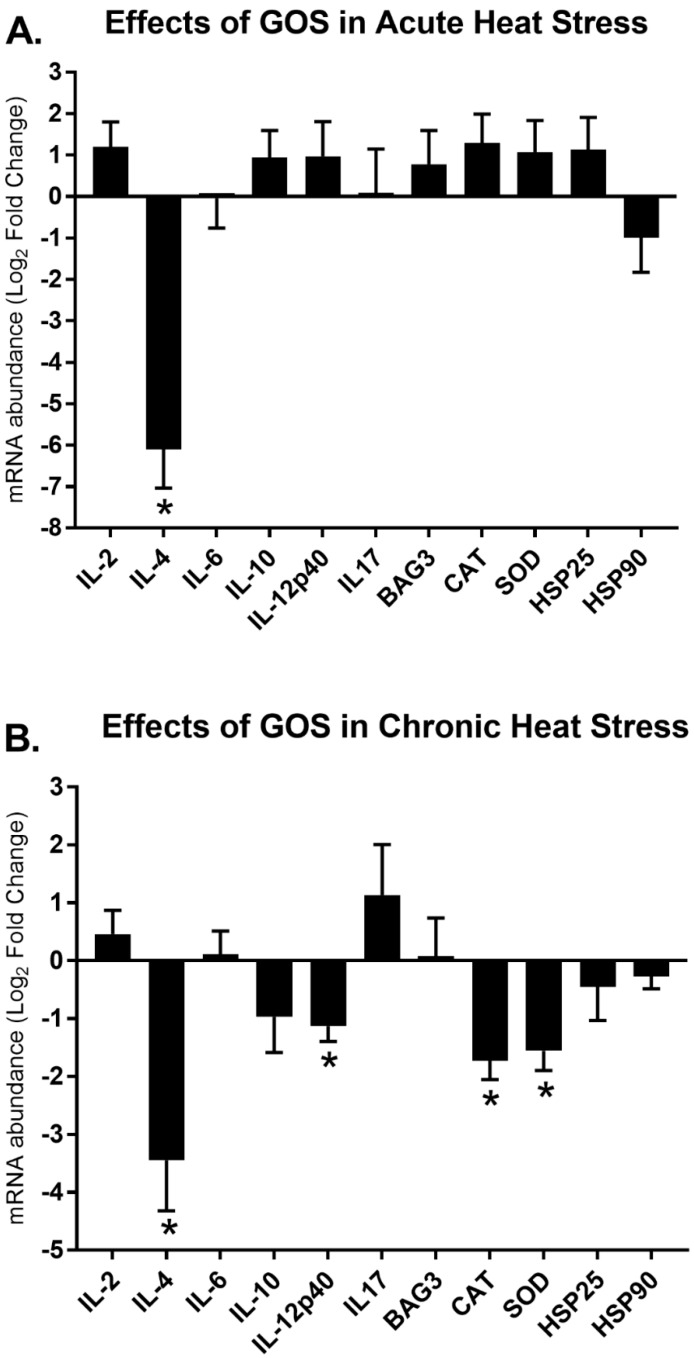
Relative mRNA expression of immune-related and stress-related genes in the spleens of slow-growing chickens injected in ovo with GOS and challenged with heat on two levels: **A**—acute (30 °C for 8.5 h); and **B**—chronic (30 °C for 14 days). Gene panel includes: interleukin: *IL-2*, *IL-4*. *IL-6*, *IL-10*, *IL-12p40*, *IL-17* and stress-related genes: CAT, SOD, BAG3, HSP25 and HSP90. X-axis shows a list of genes. Y-axis indicate relative mRNA abundance of the genes after heat challenge (n = 8). Gene expression analysis was carried out with RT-qPCR. qPCR reactions were performed in triplicates. Geometric mean of ACTB and UB reference genes was used to calculate dCt values. The relative gene expression was calculated with ddCt formula (FC = 2^−ΔΔCt^). FC values were transformed and presented as Log_2_FC. Standard error of the means (SEM) shows distribution of the Ct values. Normalized data (dCt values) of heat-challenged (A-acute, B-chronic) control (mock-injected) and heat-stressed GOS treatment groups were compared with Student’s t-test. Significant differences (*p* < 0.05) were labelled with an asterisk (*). Figures were prepared by using GraphPad Prism 7 (GraphPad, La Jolla, CA, USA).

Figure 2 presents effects of GOS that were delivered in ovo on gene expression signatures during HS. Overall, GOS that were delivered in ovo decreased the splenic expression of the immune-related and stress-related genes during HS (HS-GOS). The most striking effects of GOS that were delivered in ovo on the immune-related gene expression signatures was the down-regulation of *IL-4* cytokine during acute (FC log_2_ *IL-4* = −6.10, *p* < 0.01) and chronic (FC log_2_ *IL-4* = −3.45, *p* < 0.01) HS. Furthermore, GOS that were delivered in ovo decreased the expression of pro-inflammatory cytokine, *IL-12p40*, during chronic heat (FC log_2_ *IL-12p40* = −1.13, *p* < 0.05). Finally, in ovo treatment reduced oxidative stress induced by chronic heat (FC log_2_ CAT = −1.72, *p* < 0.05 and SOD = −1.56, *p* < 0.05). GOS that were delivered in ovo did not modulate expression of the chaperones (*p* > 0.05). 

## 4. Discussion 

### 4.1. Immune-related Gene Expression Signatures

In this study, we determined impact of two factors (i.e., the GOS that were delivered in ovo and HS) on immune-related and stress-related gene expression signatures in the spleens of slow-growing chickens. The spleen is the largest peripheral lymphoid organ and plays a key role in immune responses in chickens [50]. Gene expression signatures that were determined in the splenic tissue informed about the systemic immune response to challenging factors, including heat [51]. The GIT is highly responsive to heat. The detrimental effects of HS on intestinal homeostasis include a reduced nutrient absorption, a disrupted integrity of the intestinal wall, and an activated immune system [52,53]. Intestinal epithelial cells are connected with gap junctions, tight junctions, adherent junctions, and desmosomes [54]. Under the influence of high temperature, the barrier function is compromised, and the intestinal lumen content enters the bloodstream, causing chronic systemic inflammation [25]. The activation of Toll-like receptors (TLR) by microbial signatures triggers myeloid differentiation primary response 88 (MyD88), which in turn induces cytokine secretion [55].

Cytokines are intracellular peptides that serve as immune mediators. During HS, the levels of both pro-inflammatory and anti-inflammatory cytokines increase. This is due to endotoxemia, which is a storm of microbial endotoxin (i.e., LPS—lipopolysaccharides) infiltrating the milieu of the body [56]. In the current study, slow-growing chickens that were subjected to chronic HS demonstrated a significantly higher expression of *IL-10* and *IL-12p40*. *IL-10* skews immune responses towards Th2-type (humoral) responses, while *IL-12p40* is associated with Th1-type (cellular) immune responses [57]. There is a strong interaction between these cytokines. *IL-10* causes a negative regulation of the Th1 response [58]. In this paper, chronic (but not acute) HS increased the level of splenic mRNA encoding both *IL-10* and *IL-12p40*. There are two hypotheses of such the counter-balancing expression of pro- and anti-inflammatory cytokines in the spleens of heat-stressed chickens. First, the *IL-10* and *IL-12p40* cytokine expression in the spleen can be mediated by the TLR2 signaling pathway, triggered by Gram-positive cell wall components [59]. In heat-stressed individuals, those Gram-positive cell wall components originate from intestinal content, which leaks into the milieu of the body due to increased intestinal permeability [56]. Different bacterial ligands can stimulate TLR2 receptors on different antigen presenting cells (e.g., lymphocyte B, dendritic cells, or macrophages) in the spleen [60]. The gastrointestinal origin of those TLR ligands suggests their variability, and, as such, the ability to activate TLR signaling pathways in different cells. The second hypothesis is associated with endotoxemia that is mediated by LPS influx from the gut due to HS (as mentioned above). Endotoxemia triggers strong pro-inflammatory responses (mediated by *IL-12p40*) that are balanced by anti-inflammatory IL-10 cytokine [61]. In summary, the activation of two major cytokines in the spleens of slow-growing chickens indicates that the individuals responded to heat with increased pro- and anti-inflammatory immune responses.

GOS that were delivered in ovo balanced the level of *IL-10* and *IL-12p40* to the baseline (under acute HS) or even down-regulated cytokine expression (under chronic HS). Previously, we have determined that GOS that were delivered in ovo increased the expression of the genes that are involved in the barrier function of the gut of broiler chickens [32]. An improved barrier function allows for a decrease of intestinal permeability due to stress and the influx of antigens into the milieu of the body [62]. In the absence of an antigenic cocktail, TLR-mediated immune responses are not activated. In a broiler study that was conducted with the same experimental design as the current study, *IL-12p40* was up-regulated by acute HS, but in ovo GOS stimulation decreased its expression to the level of the control groups [41]. It can be concluded that heat induced mild immune responses in slow-growing chickens, but GOS that were delivered in ovo managed to dampen immune responses under HS.

Interleukin 4 (*IL-4*) was one of the cytokines that was modulated in slow-growing chickens by both environmental factors, i.e., GOS that were delivered in ovo and HS. Individuals that were treated with GOS under acute and chronic HS expressed a decreased mRNA level of the *IL-4* cytokine, confirmed by a significant interaction between the two factors. In our earlier study on broilers [41], the mRNA level of *IL-4* was elevated by acute HS (similarly as *IL-12p40*), but in ovo stimulation with GOS dampened its level to the baseline (i.e., mock-injected controls). In the current study on slow-growing chickens, *IL-4* was numerically down-regulated by HS alone, and the down-regulation was further enhanced by the in ovo delivery of GOS (the interaction between treatment and temperature was significant). *IL-4* cytokine acts in humoral immunity as a pleiotropic cytokine that is produced in response to receptor activation by Th2-type T cells, basophils, and mast cells [63]. Its major function is regulating antigen-stimulated naïve T cell differentiation and the expression of the specific immunoglobulin E (IgE) and immunoglobulin G (IgG) by B cells [64]. Quinteiro-Filho et al. (2017) found that chronic HS decreased the plasma levels of Immunoglobilin A (IgA) and IgG in broiler chickens [65]. It seems that splenic *IL-4* is a good biomarker of the HS response in different chicken genotypes. Early GOS delivery dampens heat-induced Th2 immune responses in slow-growing chickens.

### 4.2. Stress-related Gene Expression Signatures

Exposure to HS can lead to oxidative stress, which is characterized by the accumulation of reactive oxygen species (ROS) in the cells. The first line of defense is the production of cellular antioxidant enzymes including CAT and SOD, which protect the cells from ROS-induced cellular damage [66]. The current study showed a significant increase in the mRNA expression of *CAT* and *SOD* genes during chronic HS. Such increase indicates the activation of the pathways that are associated with counteracting the effects of oxidative stress. HS has been reported to increase the hepatic activity of CAT and SOD enzymes in two broiler chicken genotypes (Ross 308 and Cobb 500) (http://en.aviagen.com/brands/ross/products/ross-308) [67]. Habashy et al. (2018) reported the up-regulation of the mRNA level of *SOD* (but not *CAT*) gene under chronic HS (12 days) [68]. Low/basic levels of oxidative stress can play an important role in adapting to stressful environmental conditions [69]. In poultry, antioxidants use ROS to activate the expression of vitagenes, which are responsible for biological adaptation to stress. Vitagenes include the *SOD* and *HSP* genes. 

In slow-growing chickens, only chronic HS triggered mRNA responses. Long-term HS (14 days) activated anti-inflammatory (*IL-10*), pro-inflammatory (*IL-12p40*), and oxidative stress responses (*CAT* and *SOD*). Long-term HS can lead to chronic systemic oxidative stress, which is associated with mild subclinical inflammation. This condition, called “OxInflammation,” impairs natural homeostatic adaptation, which leads to stronger systemic inflammation and an increased susceptibility to diseases [70]. If it is not possible to eliminate stressors from the environment, OxInflammation can be reduced by improved acclimatization (e.g., by stimulating intestinal microbiota) and adaptation (e.g., by using slow-growing hybrids).

In this study, both *CAT* and *SOD* were up-regulated by chronic (but not acute) HS, but in ovo delivered GOS dampened the mRNA expression of both genes. GOS-stimulated chickens expressed down-regulated signatures of oxidative stress compared to mock-injected birds. Oxidative stress has been recently linked with intestinal microbiota [71]. Apparently, direct contact between intestinal epithelial cells and microbiota induces the production of physiological ROS [72]. Different species of intestinal microbiota induce different levels of ROS production in the gut. For example, *Lactobacillus* has been reported to trigger ROS production both in vitro and in vivo [72]. We previously determined that GOS that were delivered in ovo elicited bifidogenic effects in broiler chickens (i.e., a higher level of *Bifidobacteria*), which resulted in a decreased *Lactobacilli* level in the caecum [32]. We can speculate that GOS that were delivered in ovo had a potent effect on intestinal microbiota composition, which led to a decreased intestinal ROS production under chronic HS.

Splenic *HSP* genes in slow-growing chickens did not respond to HS, neither acute nor chronic. The up-regulation of *HSP* is a cellular reaction to reduce the risk of damage (by protein misfolding) during stress. In our earlier study, we observed that the mRNA expression of *HSP* was triggered in broiler chickens by acute HS, which did not cause any molecular responses in slow-growing chickens (data not presented). This suggests that slow-growing chickens are heat-tolerant, and the mild elevated ambient temperature (30 °C) did not activate HS response via chaperone proteins [73]. Comparative studies on two Brazilians native chicken breeds and commercial line Cobb chickens have shown that *HSP70* and *HSP90* gene expression differs significantly between breeds. *HSP* genes were highly up-regulated in native breeds only at a very high ambient temperature (39 °C), which proved them resistant to high temperatures [18]. 

On a performance level, HS causes losses in feed intake and growth rate. In broiler chickens, chronic HS has been found to significantly reduce (*p* < 0.01) final body weight (BW) (2.52 kg in TN vs. 3.11 kg in HS) [32,39,40]. In the current study, chronic HS also reduced final BW (*p* < 0.001), but the difference between TN and HS was almost two times lower than in broiler chickens (2.21 kg in TN vs. 1.94 kg in HS), even with the longer rearing period (42 days in broilers vs. 50 days in slow-growing chickens). The slow-growing crossbreds, which were analyzed in this study, are considered by the Hubbard breeding company as “exceptional in their rusticity.” This indicates better hardiness in comparison to highly selected and fast-growing broilers. The results presented in this paper confirm that slow-growing crossbreds lost only 12% of their total BW due to HS, whereas the losses in fast-growing broilers amounted to 24% of their total BW. GOS that were delivered in ovo improved (*p* < 0.01) the final BW in broiler chickens (2.76 kg in control chickens vs. 2.89 kg in GOS). In the current study, the final BW of slow-growing chickens was not improved (*p* > 0.05) by GOS that were delivered in ovo (2.04 kg in control chickens vs. 2.00 kg in GOS) (Federico Sirri, personal communication). The differences in growth rate seemed to have had an impact on the responses to HS in chickens. However, at this point, the data on slow-growing chickens are limited. Therefore, we find it difficult to provide an explanation for why this genotype reacted differently than the highly selected, fast-growing broilers. 

## 5. Conclusions

Slow-growing chickens proved to be well adapted to acute HS, which did not trigger immune-related or stress-related gene expression in the spleen. On the other hand, chronic HS activated genes that are associated with inflammation and oxidative stress (i.e., OxInflammation). GOS that were delivered in ovo mitigated heat-induced OxInflammation and decreased Th2 responses (down-regulation of *IL-4*). We demonstrated that the genetic adaptation of slow-growing chickens to HS combined with in ovo stimulation with GOS has mitigating effects on the molecular pathways that are associated with immune and stress responses.

## Figures and Tables

**Table 1:** Dietary formulation supplied in slow-growing chickens during three feeding phases.

Ingredient	Starter (0–14 d)	Grower (15–36 d)	Finisher (37–50 d)
Corn	42.17	34.96	12.73
White corn	0.00	0.00	15.00
Wheat	10.00	20.00	25.01
Sorghum	0.00	0.00	5.00
Soybean meal	23.11	20.63	17.60
Expanded soybean	10.00	10.00	13.00
Sunflower	3.00	3.00	3.00
Corn gluten	4.00	3.00	0.00
Soybean oil	3.08	4.43	5.48
Dicalcium phosphate	1.52	1.20	0.57
Calcium carbonate	0.91	0.65	0.52
Sodium bicarbonate	0.15	0.10	0.15
Salt	0.27	0.27	0.25
Coline cloride	0.10	0.10	0.10
Lysine solfate	0.59	0.55	0.46
Dl-methionine	0.27	0.29	0.30
Threonine	0.15	0.14	0.14
Enzyme-roxazyme g2g	0.08	0.08	0.08
Phytase 0.1%	0.10	0.10	0.10
Coccidiostat			
Vit-min premix ^1^	0.50	0.50	0.50
Dry matter, %	88.57	88.65	88.64
Protein, %	22.70	21.49	19.74
Lipid, %	7.06	8.24	9.74
Fiber, %	3.08	3.04	3.07
Ash, %	5.85	5.17	4.49
Lys, %	1.38	1.29	1.21
Met, %	0.67	0.62	0.59
Met + Cys, %	1.03	0.97	0.91
Calcium, %	0.91	0.80	0.59
Phosphate, %	0.63	0.57	0.46
Metabolizable energy (kcal/kg)	3.076	3.168	3.264

^1^ Provided the following per kg of diet: vitamin A (retinyl acetate), 13,000 IU; vitamin D3 (cholecalciferol), 4000 IU; vitamin E (DL-α_tocopheryl acetate), 80 IU; vitamin K (menadione sodium bisulfite), 3 mg; riboflavin, 6.0 mg; pantothenic acid, 6.0 mg; niacin, 20 mg; pyridoxine, 2 mg; folic acid, 0.5 mg; biotin, 0.10 mg; thiamine, 2.5 mg; vitamin B_12_ 20 μg; Mn, 100 mg; Zn, 85 mg; Fe, 30 mg; Cu, 10 mg; I, 1.5 mg; Se, 0.2 mg; and ethoxyquin, 100 mg.

**Table 2:** List of target genes and primers sequences for RT-qPCR.

Gene ^a^	NCBI Gene ID	Primer Sequences (5′-3′)	Function ^b^	Ref.
Panel 1. Immune-related genes				
*IL-2*	373958	F: GCTTATGGAGCATCTCTATCATCA R: GGTGCACTCCTGGGTCTC	Cytokine important for the proliferation of T and B lymphocytes. Important role in the immune response to antigenic stimuli.	[41]
*IL-4*	416330	F: GCTCTCAGTGCCGCTGATG R: GGAAACCTCTCCCTGGATGTC	Pleiotropic cytokine produced by activated T cells. B-cell stimulatory factor.	[43]
*IL-6*	395337	F: AGGACGAGATGTGCAAGAAGTTC R: TTGGGCAGGTTGAGGTTGTT	Cytokine that plays a role in inflammation and the maturation of B cells. Produced at sites of acute and chronic inflammation.	[44]
*IL-10*	428264	F: CATGCTGCTGGGCCTGAA R: CGTCTCCTTGATCTGCTTGATG	Pleiotropic effects in immunoregulation and inflammation. Inhibits synthesis of cytokines.	[45]
*IL-12p40*	404671	F: TTGCCGAAGAGCACCAGCCG R: CGGTGTGCTCCAGGTCTTGGG	Can act as a growth factor for activated T and Natural Killer cells. Stimulates production of IFN-gamma.	[46]
*IL-17*	395111	F: GGGATTACAGGATCGATGAGGA R: GAGTTCACGCACCTGGAATG	Cytotoxic T-lymphocyte-associated protein 8. Proinflammatory cytokine produced by activated T cells.	[41]
Panel 2. Stress response genes				
*HSP25*	428310	F: CCGTCTTCTGCTGAGAGGAGTG R: ACCGTTGTTCCGTCCCATCAC	Heat shock protein family B (small) member 9. Response to various cellular stresses. Molecular chaperones which bind to and inhibit irreversible protein aggregation or misfolding under stressful conditions.	[47]
*HSP90AA1*	423463	F: GGTGTTGGTTCCTACTCTGCTTAC R: ACTGCTCATCATCATTGTGCTTGG	Heat shock protein family class A member 1. Is a molecular chaperone that aids protein folding and quality control for a large proteins.	[47]
*BAG3*	423931	F: AGGGTCGTGCGGATGTGC R: TGTGGTGGCTTAGGCTCTGC	BAG family molecular chaperone regulator 3. Cellular response to stress.	[47]
*CAT*	423600	F: GGGGAGCTGTTTACTGCAAG R: CTTCCATTGGCTATGGCATT	Catalase a key antioxidant enzyme in the bodies defense against oxidative stress.	[48]
*SOD1*	395938	F: AGGGGGTCATCCACTTCC R: CCCATTTGTGTTGTCTCCAA	Superoxide Dismutase binds copper and zinc ions. Responsible for destroying free superoxide radicals.	[48]
Reference genes				
*ACTB*	396526	F: CACAGATCATGTTTGAGACCTT R: CATCACAATACCAGTGGTACG	Beta-actin is highly conserved protein involved in cell motility, structure, integrity and intercellular signaling. Ubiquitously expressed in all eukaryotic cells.	[49]
*UB*	101747587F	F: GGGATGCAGATCTTCGTGAAA R: CTTGCCAGCAAAGATCAACCTT	Ubiquitin is associated with protein degradation, DNA repair, cell cycle regulation kinase modification, and regulation of other cell signals pathways.	[49]

^a^ Annealing temperature for RT-qPCR was 58 °C except from *IL-12p40* (65 °C); ^b^ gene function derive from GeneCards (http://www.genecards.org).

**Table 3:** Effects of in ovo treatment and ambient temperature on gene expression signatures in the spleens of slow-growing chickens.

Gene	Treatment ^1^	Temperature ^2^	Treatment × Temperature ^3^
Acute HS			
Immune-related panel			
*IL-2*	NS	NS	NS
*IL-4*	<0.001	<0.05	<0.01
*IL-6*	NS	NS	NS
*IL-10*	NS	NS	NS
*IL-12p40*	NS	NS	NS
*IL-17*	NS	NS	NS
Stress-related panel			
*BAG3*	<0.05	NS	NS
*CAT*	<0.05	NS	NS
*SOD*	<0.05	NS	NS
*HSP25*	NS	NS	NS
*HSP90*	NS	NS	NS
Chronic HS			
Immune-related panel			
*IL-2*	<0.05	NS	NS
*IL-4*	<0.001	<0.01	NS
*IL-6*	NS	NS	NS
*IL-10*	NS	NS	NS
*IL-12p40*	NS	<0.05	NS
*IL-17*	NS	NS	NS
Stress-related panel			
*BAG3*	NS	NS	NS
*CAT*	NS	NS	<0.01
*SOD*	NS	NS	<0.01
*HSP25*	NS	NS	NS
*HSP90*	NS	NS	NS

Effects: ^1^ In ovo delivery of galactooligosaccharides (GOS) vs. physiological saline (C); ^2^ ambient temperature (TN—thermoneutral vs. HS); ^3^ interaction between in ovo treatment and ambient temperature on immune-related and stress-response genes in chicken spleens. Gene expression analysis was done with RT-qPCR. The significance of effects that were calculated with two-way ANOVA. Significance levels: *p* < 0.05, *p* < 0.01 or *p* < 0.001 (significant), and *p* > 0.05 (non-significant, NS).

**Table 4:** Effects of in ovo treatment and the duration of heat stress (HS) on gene expression signatures in the spleens of slow-growing chickens.

Gene	Treatment ^1^	HS ^2^	Treatment × HS ^3^
Immune-related panel			
*IL-2*	NS	<0.05	NS
*IL-4*	<0.001	NS	NS
*IL-6*	NS	NS	NS
*IL-10*	NS	NS	NS
*IL-12p40*	NS	NS	NS
*IL-17*	NS	NS	NS
Stress-related panel			
*BAG3*	NS	NS	NS
*CAT*	NS	NS	<0.05
*SOD*	NS	NS	<0.05
*HSP25*	NS	NS	NS
*HSP90*	NS	<0.05	NS

Effects: ^1^ In ovo delivery of galactooligosaccharides (GOS) vs. physiological saline (C); ^2^ HS (acute HS vs. chronic HS); ^3^ interaction between in ovo treatment and ambient temperature on immune-related and stress-response genes in chicken spleens. Gene expression analysis was done with RT-qPCR. The significance of effects was calculated with two-way ANOVA. Significance levels: *p* < 0.05, *p* < 0.01 or *p* < 0.001 (significant), and *p* > 0.05 (non-significant, NS).
